# Habitat fragmentation influences genetic diversity and differentiation: Fine‐scale population structure of *Cercis canadensis* (eastern redbud)

**DOI:** 10.1002/ece3.6141

**Published:** 2020-03-16

**Authors:** Meher A. Ony, Marcin Nowicki, Sarah L. Boggess, William E. Klingeman, John M. Zobel, Robert N. Trigiano, Denita Hadziabdic

**Affiliations:** ^1^ Department of Entomology and Plant Pathology University of Tennessee Knoxville TN USA; ^2^ Department of Plant Sciences University of Tennessee Knoxville TN USA; ^3^ Department of Forest Resources University of Minnesota St. Paul MN USA

**Keywords:** *Cercis canadensis*, fine‐scale population structure, genetic diversity, habitat fragmentation, redbud

## Abstract

Forest fragmentation may negatively affect plants through reduced genetic diversity and increased population structure due to habitat isolation, decreased population size, and disturbance of pollen‐seed dispersal mechanisms. However, in the case of tree species, effective pollen‐seed dispersal, mating system, and ecological dynamics may help the species overcome the negative effect of forest fragmentation. A fine‐scale population genetics study can shed light on the postfragmentation genetic diversity and structure of a species. Here, we present the genetic diversity and population structure of *Cercis canadensis* L. (eastern redbud) wild populations on a fine scale within fragmented areas centered around the borders of Georgia–Tennessee, USA. We hypothesized high genetic diversity among the collections of *C. canadensis* distributed across smaller geographical ranges. Fifteen microsatellite loci were used to genotype 172 individuals from 18 unmanaged and naturally occurring collection sites. Our results indicated presence of population structure, overall high genetic diversity (*H*_E_ = 0.63, *H*_O_ = 0.34), and moderate genetic differentiation (*F*_ST_ = 0.14) among the collection sites. Two major genetic clusters within the smaller geographical distribution were revealed by STRUCTURE. Our data suggest that native *C. canadensis* populations in the fragmented area around the Georgia–Tennessee border were able to maintain high levels of genetic diversity, despite the presence of considerable spatial genetic structure. As habitat isolation may negatively affect gene flow of outcrossing species across time, consequences of habitat fragmentation should be regularly monitored for this and other forest species. This study also has important implications for habitat management efforts and future breeding programs.

## Graphical abstract

In this study, we used microsatellite loci to estimate genetic diversity of *Cercis canadensis* L. (eastern redbud) wild populations on a fine scale. Our data reveal that current isolated patches and remnant populations are genetically stable and capable of maintaining viable populations at a geographically fine‐scale level. Forest fragmentation may cause a number of negative effects including, but not limited to, reduced genetic diversity and population size, increased population structure due to habitat isolation, and potential disruption of pollen‐seed dispersal.

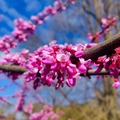

## 1 INTRODUCTION

Habitat fragmentation involves discontinuities in the distribution of an organism due to geographical and/or geological barriers, and/or human activities (Kolb & Diekmann, 2005; Kwak, Velterop, & Andel, 1998). For example, roadways have transected forests and imposed long, linear artificial structure to forested patches that, in turn, may be invaded by a mix of ornamental plants, ornamental plant cultivars, and their wild‐type progeny (Hamberg, Lehvävirta, & Kotze, 2009; Hardiman & Culley, 2010; Kwak et al., 1998). Such fragmentation impacts the genetic diversity and population structure of a species in various ways depending on the ecology and biology of the species (Cuartas‐Hernández & Núñez‐Farfán, 2006; Suárez‐Montes, Chávez‐Pesqueira, & Núñez‐Farfán, 2016). In a constantly changing natural setting, the dynamics of a plant population and ecological succession of plant species are shaped by the following three major challenges: species dispersal, establishment, and persistence in a specific environment (Weiher et al., 1999).

Habitat fragmentation may affect these processes through a number of ecological and biological modifications imposed on the fragmented area (Haila, 1999; Kolb & Diekmann, 2005; Suárez‐Montes et al., 2016). For instance, pollination‐ and animal‐based seed dispersal mechanisms are negatively impacted by forest fragmentation, especially at local levels (Dickson, 1990; Santos, Tellería, & Virgós, 1999; Sato & Kudoh, 2014). Disruptions in dispersal processes can reduce gene flow and increase inbreeding within these spatially isolated populations (Kearns, Inouye, & Waser, 1998; Van Geert, Rossum, & Triest, 2007). Loss of habitat, or even its degradation caused by fragmentation, can reduce the availability of suitable habitat and, therefore, negatively influence species establishment (Haila, 1999; Kolb & Diekmann, 2005). Finally, habitat fragmentation can lead to reduction in population size that results in reduced genetic variations, adaptive potential, and survival of the members in the smaller, isolated population (Sherwin & Moritz, 2000; Van Geert et al., 2007). Additionally, fragmentation reduces the natural habitat of a species, but also creates artificial edges that differ in plant composition from the rest of the forested area (Hamberg et al., 2009). The presence of forest edges may increase the habitat area for different ecological niches typically found in higher numbers in these types of ecosystems (Hamberg et al., 2009).

In many cases, tree species in temperate forests are less likely to be impacted by genetic consequences of forest fragmentation compared to tropical tree species (Kramer, Ison, Ashley, & Howe, 2008). This outcome is partly explained by the higher tree density and undisturbed pollen‐seed dispersal. This can, in turn, ensure sufficient gene flow across these isolated populations, thus reducing the potential threat of genetic declines to temperate tree species in North America (Byrne, Elliott, Yates, & Coates, 2008; Kramer et al., 2008; Nason, Herre, & Hamrick, 1998). Several economically and socially important tree species that have been extensively exploited as a result of logging and forest fragmentation have shown little to no genetic consequences of these demographic events challenging their sustainability (Marquardt, Echt, Epperson, & Pubanz, 2007; Victory, Glaubitz, Rhodes, & Woeste, 2006). In contrast, *Taxus baccata* L., a forest tree in Spain, was negatively affected by chronic fragmentation and revealed strong spatial structure with a recent bottleneck history in spite of abundant seed dispersal mechanisms (Dubreuil et al., 2010). Fragmented systems can result in a reduction in overall species health that can in turn restructure forest compositions in impacted communities and disrupt the ecosystem processes of tree species (Hall, Motzkin, Foster, Syfert, & Burk, 2002). Since the European settlement of the United States, more than 220 plant species have become extinct in North America and Hawaii (Noss, LaRoe, & Scott, 1995; Russell & Morse, 1992). Moreover, in the list of threatened and endangered species in the United States, 81% were affected by anthropogenic activities (Cook & Dixon, 1989; Noss et al., 1995).

In geographically reduced or fragmented populations, genetic diversity level can be affected through genetic drift and inbreeding, which can additionally reduce the ability of the affected individuals to regenerate and respond to changes of selection pressures (Hadziabdic et al., 2010; Suárez‐Montes et al., 2016). Genetic drift and inbreeding in a plant species can erode the overall population fitness and the prospects for adaptive change, thus increasing the possibility of species decline, mortality, or extinction (Fischer & Matthies, 1998; Severns, 2003; Young, Boyle, & Brown, 1996). Due to constantly changing forest structures as a result of fragmentation, urbanization, and environmental conditions, knowledge of current genetic diversity and spatial structure of economically and/or environmentally important species is often unknown or limited. Here, we focused on the U.S. native *Cercis canadensis* L. (eastern redbud) wild or naturally occurring populations distributed across a smaller, fragmented geographical area around the Georgia and Tennessee border, USA.

**Figure 1:**
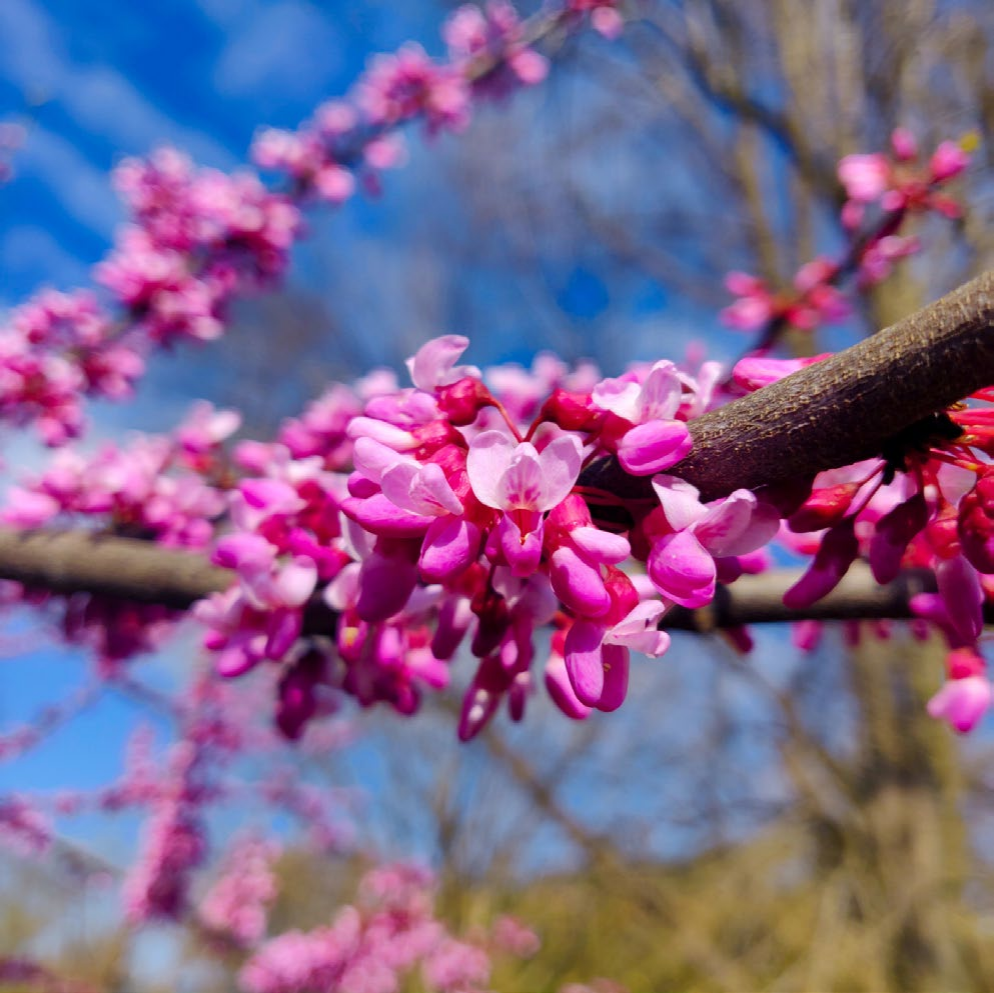
Flowers of *Cercis canadensis* in full bloom. Ramiflorous flowers of *C. canadensis* emerge in clusters directly from beneath bark on bare branches with flowering occurring prior to expansion of juvenile leaves

**Figure 2:**
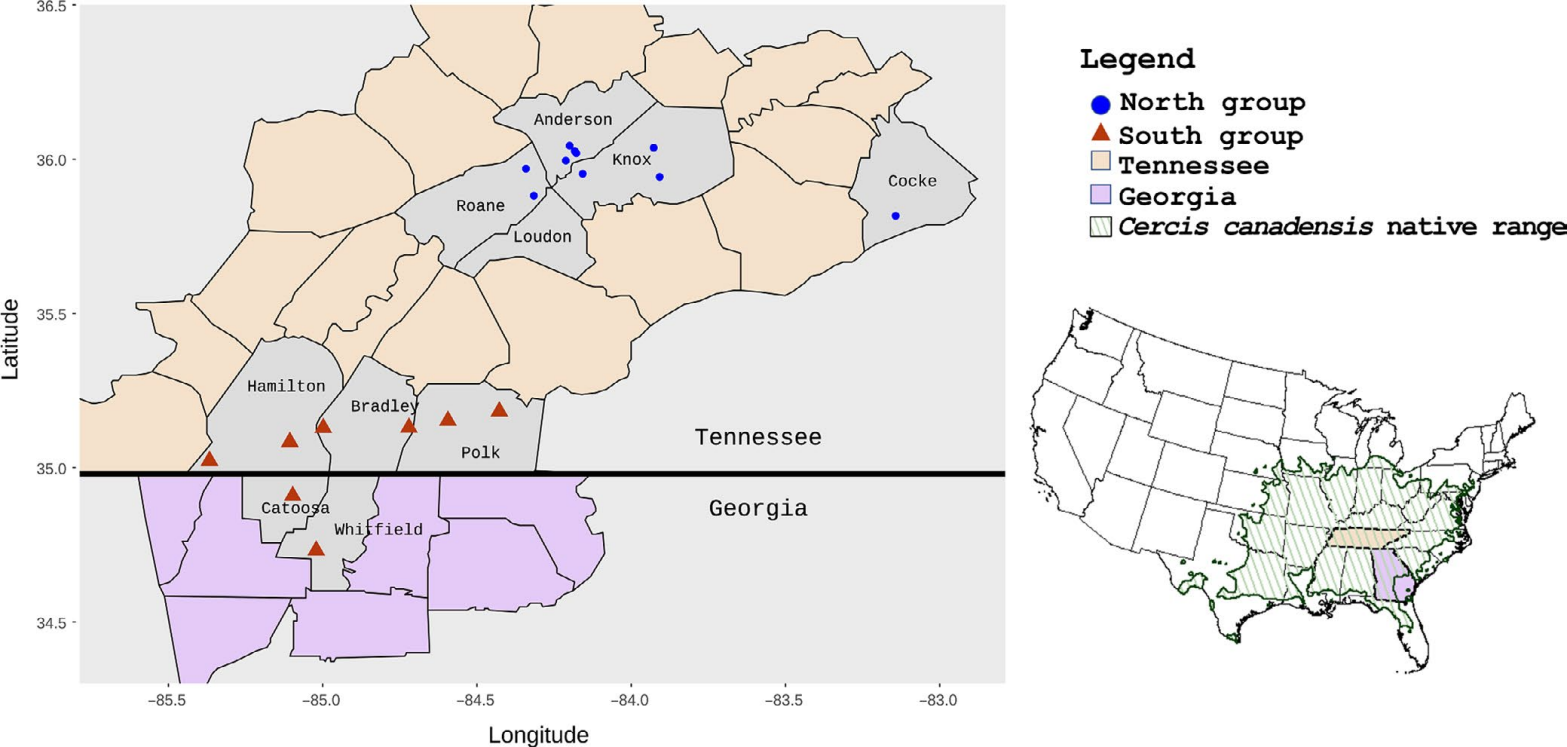
Geographical distribution of *Cercis canadensis* in the eastern United States (insert) and map of 18 collection sites used in this study

To address the effect of fragmentation, in this study we used a native, understory tree *Cercis canadensis* (eastern redbud; Figure 1). The tree is widely distributed across the eastern United States and extends into the northern part of central Mexico (Figure 2; Couvillon, 2002; Davis, Fritsch, Li, & Donoghue, 2002; Dirr, 1990). *Cercis* *canadensis* is a small‐to‐medium‐size ornamental tree that presents an umbrella‐shaped crown and foliage that exhibits varying colors across the season that ranges from deep purple, green to yellow (Pooler, Jacobs, & Kramer, 2002; Trigiano, Beaty, & Graham, 1988; Wadl, Trigiano, Werner, Pooler, & Rinehart, 2012). Characteristic heart‐shaped leaves, wide range of foliage and floral colors, and early spring blooms make *C. canadensis* a popular ornamental landscape tree in the temperate North America (Figure 1; Dickson, 1990; Wadl et al., 2012). There are more than three dozen commercial cultivars of *C. canadensis* and other *Cercis* species currently available in retail and wholesale trade (Thammina, Kidwell‐Slak, Lura, & Pooler, 2017; Wadl et al., 2012). Consequently, *Cercis* spp. cultivars have achieved an annual U.S. market value of 27 million USD (USDA, 2014).

In addition, *C. canadensis* is well adapted to mesic, semiarid to sometimes in xeric environments. This self‐incompatible tree can grow as a shade‐tolerant understory tree in closed forests (the mid‐ to deep southern United States), open woodlands, and forest borders (northern region of the species distribution) in full sunlight (Abram, 1986; Griffin, Ranney, & Pharr, 2004; Norcini, Knox, & Andersen, 1991; Pooler et al., 2002). The tree is commonly found on southward slopes with full sunlight and open forest edges where there are low levels of competition with other tree species. Also, *C. canadensis* tolerates a wide range of soil types and pH throughout the species’ wide geographical distribution (Dickson, 1990). *Cercis canadensis* is self‐incompatible; therefore, it relies on pollen and potentially animal‐mediated seed dispersal to enable reproduction (Roberts, Werner, Wadl, & Trigiano, 2015). Currently, our knowledge regarding the effects of forest fragmentation at local levels on the genetic diversity and spatial distribution of wild populations of *C. canadensis* is limited.

Given the popularity of this species as a landscape specimen, we investigated genetic diversity and spatial structure of wild *C. canadensis* across a small geographical area in eastern Tennessee and around the Tennessee–Georgia border in the eastern United States. Over the last century, forest fragmentation has increased in this area because of regional urbanization and human infrastructure development (Lloyd, 2012; Zhang et al., 2012). Our hypothesis was that despite increased habitat fragmentation, wild populations of *C. canadensis* at local levels would have high genetic diversity with the presence of population structure and moderate‐to‐high gene flow. To test this hypothesis, previously developed microsatellite loci (Wadl et al., 2012) were used to achieve the following objectives: (a) to evaluate the fine‐scale genetic diversity present within *C. canadensis* populations occurring in eastern Tennessee and along the Georgia–Tennessee border and (b) to infer fine‐scale patterns in the spatial distribution and gene flow of *C. canadensis* Tennessee and on the Georgia–Tennessee border.

## 2 MATERIALS AND METHODS

### 2.1 Sample collection

Samples of *C. canadensis* were collected from eastern Tennessee and around the Georgia–Tennessee border, USA, which is located near the center of the current native distribution range of the species (Figure 2) and not far from The University of Tennessee, Knoxville laboratory (35°56′53.8″N; −83°56′28.9″W). Young and newly expanded leaves from 10 to 12 noncultivated (wild) trees per collection site were collected at 18 locations (Table S1; *n* = 180 trees). From each tree, five to seven leaves were randomly collected from branches that were at cardinal directions around each tree. Leaves were placed in paper envelopes to dry and geographical coordinates were recorded for each sampled tree. Samples collected from each location were considered a single collection site.

### 2.2 DNA extraction

DNA was isolated from approximately 100 mg of dried leaf tissue. Samples were homogenized four times using a Bead Mill 24 (Fisher Scientific) with the settings of *S* (speed) = 6.00 m/s, *T* (time) = 30 s. Between each homogenization step, samples were frozen in liquid nitrogen for 5 min to improve the tissue homogenization. Genomic DNA (gDNA) from each sample was isolated using the Qiagen DNeasy Plant Mini Kit (Qiagen) using the manufacturer's protocol with several modifications, which included the following: 2% w/v polyvinylpyrrolidone (PVP) was mixed into lysis buffer (AP1); 8 µl of RNase was added into each sample tube; the time period of incubation at 65°C was increased to 45 min and inverted gently to mix the sample every two minutes; incubation step at −20°C was followed with increased time of incubation to one hour. Additionally, before adding the elution buffer, ethanol was used to wash the spin columns if there was any debris left from the sample tissue. Finally, 50 μl of elution buffer preheated at 65°C was added, and the step was repeated twice, for a total elution of 100 μl. The quality and concentration of the isolated gDNA was assessed using ND1000 Visible (UV–Vis) Spectrophotometer (NanoDrop Technologies, Wilmington, DE, USA).

### 2.3 Microsatellite primers and genotyping conditions

Primers for 68 genomic microsatellite loci (Wadl et al., 2012) were synthesized by Integrated DNA Technologies (IDT). Because the 68 primers were developed for a cross‐species amplification study among eight species representing the genus *Cercis* and another closely related species *Bauhinia faberi* Oliver (Wadl et al., 2012), further screening was needed to optimize these primers for the wild *C. canadensis* samples used in this study. We used gDNA samples of five *C. canadensis* individuals from the University of Tennessee (UTK) Gardens to screen all of the primers used in this study. From our initial screening of the 68 microsatellite loci, 15 primer pairs were selected based on polymorphic loci, as well as a successful amplification rate across the five tested samples (Table 1).

**Table 1:** Genetic diversity indices across 18 collection sites of *Cercis canadensis* using 15 microsatellite loci

Locus	Genbank accession no.	Forward and reverse primers (5′−3′)	Repeat motif	Size range (bp)	No. of alleles	Ar	*H*_o_	*H*_e_	*H*	*F*_ST_	*F'*_ST_	*F*_IS_	Nm
1057a	GU171393	F:TCCCTCTCAGCTTTCATATAATCCAC R:AAAGAGAGATCGTTTAGAAGGCGG	(CCATCA)_7_	116–175	11	5.90	0.82	0.84	2.00	0.05	0.05	−0.03	2.49
127spa	GU252892	F:CCAATTCAATTCCTCTGTGTGTTG R:AACGGTGTGACTAGGAGTCAAAGG	(TC)_4_	87–112	7	4.92	0.98	0.76	1.17	0.04	0.05	−0.34	1.75
168a	GU252915	F:AACAAAAGCAAAAGCACGCTACTC R:CAGTTGCCAAAATCAGAGAAATTG	(CT)_7_	151–175	6	1.78	0.04	0.47	0.83	0.59	0.61	0.80	0.15
177b	GU252919	F:AGAAATTTCAGAGACCGTGAGGTG R:TAACACACTATCCGTCATTCCCAG	(GA)_6_	154–183	5	3.34	0.43	0.67	1.22	0.04	0.04	0.33	2.24
199a	GU252924	F:AATAACTCCTGGAACAATGGAGGG R:TCTATGGTTTAGACCCTTTGTCACATC	(GAGA)_8_	149–173	6	2.95	0.25	0.51	1.02	0.12	0.12	0.45	1.12
220a	GU252932	F:ACCCATTCACTACCGTTCATTGAG R:GATTCCAGATTGTCACACGTTTTG	(TATT)_4_	100–123	7	3.68	0.67	0.68	1.30	0.22	0.23	−0.26	0.79
229a	GU252937	F:CTGAGGTCCGAATGGTAATTGAAC R:CGATAATACTCGATATATGCATTGCG	(GAGAG)_4_	147–170	4	2.17	0.29	0.30	0.58	0.03	0.03	0.01	3.07
53a	GU252855	F:TCCTTTGCTCATGGTAGTCTGATG R:GCACTAAAGAGTTGTGTTCATGCC	(AAAT)_6_	128–165	10	5.23	0.25	0.75	1.66	0.07	0.07	0.65	1.33
625a	GU253092	F:TTGTGGTTCTAGCCTTTGCTTTTC R:GCACTAAAGAGTTGTGTTCATGCC	(GA)_4_	95–142	5	3.73	0.21	0.68	1.03	0.18	0.19	0.62	0.60
658a	GU253101	F:TTTTCAGAGCGTTATCACTCAACG R:CCCTAAGTAGGAGCACTCCTTTCC	(CT)_6_	97–123	5	3.34	0.23	0.54	0.97	0.10	0.11	0.52	0.84
680a	GU253111	F:AAATTTAAAGACCCCATTGCCAAC R:ACACTCCCACAAAACCTTCACTTC	(GT)_8_	144–152	5	2.24	0.00	0.60	1.01	0.33	0.34	1.00	0.40
762a	GU253134	F:TCTGTCTCACCTGCTTGCACTAAG R:GGCTCAATCTCCAAGAAAATGAAG	(TC)_7_	94–114	7	3.00	0.06	0.65	1.19	0.07	0.07	0.90	1.32
780b	GU253139	F:TAGAGCCCTATTCCCACTTGACAC R:CTTTATGAATGGTTGTCTTGCTGG	(AG)_12_	142–175	12	5.71	0.79	0.80	1.88	0.04	0.04	−0.03	2.34
871a	GU253176	F:TTCTTAAGCTAAACGGTGCATTTTG R:GATGAGGGTTGGTGTAGTGAGGAG	(CTT)_9_	112–159	12	4.15	0.13	0.70	1.58	0.07	0.07	0.80	1.03
995a	GU253208	F:GTGCTTTGTCTTTGTGTTCCATTC R:AAAACTACGCGTCCCTTCCTTC	(AG)_7_	109–127	4	1.88	0.00	0.46	0.86	0.43	0.44	1.00	0.27
Average					7	3.60	0.34	0.63	1.22	0.14	0.15	0.36	1.32

Note

Ar, allelic richness corrected for sample size; *F*_ST_, population fixation index; *F'*_ST_, population differentiation; *F*_IS_, inbreeding coefficient; *H*, Shannon–Wiener index; *H*_o_, observed heterozygosity; *H*_e_, Nei's genotypic diversity; Nm, gene flow.

Polymerase chain reaction (PCR) amplifications were completed in 10 µl reaction mixture consisting of the following: 1 µl undiluted gDNA, 1 µl of 10 µM of each forward and reverse primer, 0.5 µl of dimethyl sulfide, 4 µl of GoTaq G2 Hot Start Master Mix (Promega Corp), and 2.5 µl water. Both positive control (a DNA sample that amplified across all microsatellite loci) and negative control (control reaction without any DNA sample) samples were used for every primer tested to ensure validity of the data. Amplification of reactions was completed in 96 well plates using an Eppendorf thermocycler (Eppendorf AG) with the following thermal profile: initial denaturation at 94°C for 3 min, followed by 35 cycles of denaturation at 94°C for 30 s, annealing at 55°C for 30 s. and an extension at 72°C for 30 s, with a final extension of 72°C for 4 min. Amplified PCR products were visualized using a QIAxcel Capillary Electrophoresis System (Qiagen) and sized with a 15/600 bp internal alignment marker and a 25 to 500 bp DNA size marker. All 180 *C. canadensis* gDNA samples were tested against each of the 15 microsatellite loci using the procedure described above. Failed reactions were repeated twice before considering them as missing data in the dataset.

### 2.4 Genetic diversity

The FLEXBIN Excel macro version 2 (Amos et al., 2007) was used to bin raw alleles into statistically similar allelic classes. The binned allelic data were used for all statistical analyses. Samples were divided into the following two groups based on geographical distance from each other: the 10 collection sites from eastern Tennessee were combined in one group (north group) and the eight collection sites from the Georgia–Tennessee border into the second group (south group; Table S1). To avoid overrepresentation of possible clonal samples in the dataset (e.g., originating from a planted cultivar, rather than a pollinated, wild‐type tree), clone correction was completed using POPPR version 2.8.2 (Kamvar, Tabima, & Grünwald, 2014) in R (R Core Team, 2019) version 3.5.3. Only unique multilocus genotypes (MLGs) per collection site were used for further analyses so that unbiased allele frequency estimates could be obtained (Tsui et al., 2012). Eight samples were discarded due to missing data in more than 25% of loci resulting in 172 samples for all subsequent analyses.

Genetic diversity indices across 15 microsatellite loci and 18 collection sites of *C. canadensis* were calculated using package POPPR. For each microsatellite locus, the number of alleles, observed heterozygosity (*H*_o_; calculated as the number of the individual heterozygotes present at a locus divided by sample size), and expected heterozygosity (*H*_e_; expected heterozygosity per tested locus (Nei, 1978)) were estimated. Allelic richness (Ar; a measure of rarefied allelic counts per locus) is an estimation of the long‐term potential of a population to adapt and persist in a given population, was calculated using package hierfstat (El Mousadik & Petit, 1996; Hurlbert, 1971; Petit, Mousadik, & Pons, 1998). Presence of unique private alleles in different loci and collection sites was also calculated using package POPPR. Genetic fixation index (*F*_ST_; a measure of nearness to allelic fixation within a population), genetic differentiation (*F′*_ST_, a measure of relative degree of differentiation in allele frequency; Bird, Karl, Smouse, & Toonen, 2011; Jost, 2008; Jost et al., 2018), and inbreeding coefficient (*F*_IS_; an estimator of the probability that two alleles at a random locus are from the same ancestor (Wright, 1990)) were also calculated using package hierfstat. In addition, the Shannon–Wiener diversity index (*H*) was estimated which combines both allele richness and evenness of the allele distribution (Shannon, 1948). *H* index value increases with increased richness and evenness. Moreover, as *H* is a logarithmic function, exp(*H*) will provide the number of expected alleles that are evenly distributed in the studied dataset (Grünwald, Goodwin, Milgroom, & Fry, 2003). Gene flow (Nm) among collection sites was estimated using GenAlEx 6.5 software (Peakall & Smouse, 2006, 2012) and was calculated as number of effective migrants per locus on the basis of *F*‐statistics. Also, pairwise *F*_ST_ among 18 collection sites were estimated using the R package adegenet version 2.1.1 (Jombart & Ahmed, 2011).

### 2.5 Population structure

Population structure of the *C. canadensis* collection was analyzed with the program STRUCTURE version 2.3.4 (Pritchard, Stephens, & Donnelly, 2000) by utilizing Monte Carlo Markov chain (MCMC) using a Bayesian method of clustering. To infer genetic clusters among *C. canadensis* individuals, the following parameters were used: 500,000 burn‐in period with 500,000 MCMC repetitions using 20 independent chains for each *K* value (number of inferred population clusters from 1 to 18). Results were analyzed with STRUCTURE HARVESTER Web version 06.94 (Earl, 2012). The optimum value of *K* was estimated using the Evanno method (Evanno, Regnaut, & Goudet, 2005). For STRUCTURE analyses, we used an admixture model to interpret the proportion of individuals originating from different groups or clusters. The results of the Δ*K* criterion attained from STRUCTURE HARVESTER were visualized using POPHELPER 2.2.6 (Francis, 2017).

However, the Evanno method used by STRUCTURE HARVESTER is unable to calculate a value of Δ*K* less than two. To mitigate this issue and ensure the accuracy of our STRUCTURE results, we utilized the program InStruct, a Bayesian clustering method used to infer population structure (Gao, Bryc, & Bustamante, 2011; Gao, Williamson, & Bustamante, 2007). This program considers the presence of clonal populations (*K* = 1) and disregards the assumption of Hardy–Weinberg equilibrium within groups of populations. The following parameters were used in this analysis: 20 independent MCMC chains for each *K* value (1–18) with a burn‐in period of 500,000 and 500,000 repetitions of the thinning interval using the admixture model. An admixture model was implemented to evaluate the proportion of mixed ancestry in an individual and improve the clustering (Lind and Gailing, 2013). Deviance information criterion (DIC) values were estimated to obtain an optimum *K* value (Gao et al., 2011).

Two model‐free clustering approaches were implemented to delineate the population clusters. Nei's genetic distance was used to construct a neighbor‐joining dendrogram. Discriminant analysis of principal components (DAPC), a model‐free multivariate analysis approach, is a useful tool to investigate and visualize the presence of genetic clusters (Jombart, Devillard, & Balloux, 2010). The DAPC method was implemented and visualized with the R package adegenet, which defines discriminant axes along which groups can be separated. The DAPC was optimized with 35 of retained PCs (PCA eigenvalues) and cross‐checked with 1,000 permutations of the dataset. For this analysis, missing values were treated as mean allele frequency, and 90% of samples from each collection site were used to run cross‐validation analysis to determine the appropriate number of PCs.

Analysis of molecular variance (AMOVA; Excoffier, Smouse, & Quattro, 1992) was estimated using POPPR with 9,999 permutations by grouping the individuals into three hierarchical groups to evaluate the molecular variance partitioning within and among the collection sites. The levels of population hierarchy were as follows: (a) among groups suggested by the STRUCTURE analysis, and (b) among the geographically separate north and south groups.

To estimate the isolation by distance (IBD), a Mantel test (Diniz‐Filho et al., 2013; Mantel, 1967) was completed with 10,000 permutations using the MASS package version 7.3‐51.1 (Venables & Ripley, 2002) in R using Euclidean distance. IBD discerns whether or not there is correlation between genetic and geographical distance of the studied individuals. The Mantel test was run across 18 collection sites by considering them as one population, as well as across the two collection sites (north and south).

### 2.6 Demography

The program BOTTLENECK version 1.2.02 (Cornuet & Luikart, 1996) was implemented to investigate the evidence of a recent bottleneck. To test whether a recent bottleneck or expansion of *C. canadensis* populations on a fine geographical scale had occurred, two mutation models were utilized: stepwise‐mutation model (S.M.M.), infinite allele model (I.A.M.), and two‐phase mutational model (T.P.M.). Significance of this test under either of these models was evaluated by the Wilcoxon sign‐rank test with 10,000 simulations, as the number of loci for this study was under 20 (Cornuet & Luikart, 1996; Piry, Luikart, & Cornuet, 1999).

## 3 RESULTS

### 3.1 Microsatellite genetic diversity and hierarchical fixation indices

For data analyses, genotypic data of 172 individuals were used. After clone correction, all 172 samples resulted in unique multilocus genotypes. Therefore, the results presented here include a total of 172 *C. canadensis* individuals from Tennessee and Georgia without the presence of clonal samples in the dataset. An average of 1.94% missing data were detected across the dataset (Figure S1). Locus 871a contained the highest number of missing values (13.95%) whereas collection site Knox Co.1 had highest number of missing values (7.42%) among the 18 collection sites. An average of seven alleles (ranged from four to 12) per locus were identified (Table 1). Mean allelic richness (Ar) was 3.60, ranging from 1.78 for locus 168a to 5.90 for locus 1057a, which demonstrated high allelic richness among *C. canadensis* individuals. The overall observed heterozygosity across 15 microsatellite loci was 0.34, ranging from 0.00 (680a and 995a) to 0.98 (127spa). The overall expected heterozygosity (*H*_e_) across all 15 microsatellite loci was high (*H*_e_ = 0.63), ranging from 0.30 (locus 229a) to 0.84 (locus 1057a). Additionally, the overall Shannon–Wiener diversity index (*H*) was 1.22 for the 15 loci ranging from 0.58 (locus 229a) to 2.00 (locus 1057a; Table 1). Furthermore, our data indicated a moderate‐to‐high population fixation value (*F*_ST_ = 0.14; ranging from 0.03 to 0.59; Table 1), high population differentiation (*F*′_ST_ = 0.15; ranging from 0.03 to 0.61; Table 1), and presence of inbreeding (*F*_IS_ = 0.36; Table 1) among *C. canadensis* collection sites across the 15 microsatellite loci. High gene flow was also detected among the studied population with an average of 1.32 across 15 microsatellite loci.

The accrued MLG dataset was analyzed in the following two settings: (a) considering all the 18 collection sites as one population and (b) dividing the entire dataset into two groups, where the first group (north group) represented collection sites from eastern Tennessee and the second group (south group) represented the collection sites near the Georgia–Tennessee border (Figure 2).

Private alleles (*n* = 15) were detected in 10 out of 18 collection sites (Table 2). When data were partitioned into two collection sites, 33 private alleles were detected (16 and 17 across the north and south groups, respectively; Table 2). For the 18 collection sites, Nei's genetic diversity index (*H*_e_), corrected for the collection site sample size, ranged from 0.33 (Polk Co. 1) to 0.62 (Bradley Co. 2), with an overall value of 0.61 which indicates presence of high genetic diversity in the studied dataset. Comparably, in the two‐group analysis, the north group had lower overall genetic diversity when compared to the south group (*H*_e_ = 0.53 and *H*_e_ = 0.61, respectively; Table 2). Also, pairwise *F*_ST_ based on Nei's genetic distance of 18 collection sites ranged from 0.02 (among the collection sites Bradley Co. 2, TN, Catoosa Co., GA and Whitfield Co., GA) to 0.31 (between the collection sites Polk Co. 1, TN and Hamilton Co. 1, TN; Table S2). All of these five collection sites belonged to the south group.

**Table 2:** Genetic diversity indices of (a) 18 collection sites and (b) two collection sites of *Cercis canadensis* using 15 microsatellite loci

(a) 18 collection sites					
Collection site name	Group	*N*	MLG	*H*_e_	Pa
Anderson Co.1	North Group	10	10	0.49	1
Knox Co.1	North Group	7	7	0.51	0
Loudon Co.	North Group	10	10	0.44	0
Roane Co.	North Group	9	9	0.44	0
Anderson Co.2	North Group	7	7	0.53	0
Anderson Co.3	North Group	10	10	0.46	0
Anderson Co.4	North Group	10	10	0.41	2
Cocke Co.	North Group	10	10	0.50	1
Knox Co.2	North Group	10	10	0.51	2
Knox Co.3	North Group	9	9	0.47	0
Polk Co.1	South Group	10	10	0.33	1
Polk Co. 2	South Group	10	10	0.46	2
Bradley Co.1	South Group	10	10	0.54	2
Hamilton Co.1	South Group	10	10	0.49	0
Hamilton Co.2	South Group	10	10	0.58	2
Bradley Co.2	South Group	10	10	0.62	0
Catoosa Co.	South Group	10	10	0.61	1
Whitfield Co.	South Group	10	10	0.58	1
Total/average		172	172	0.61	15

(b) Two Geographical groups				
Subpopulations	*N*	MLG	*H*_e_	Pa
North group	92	92	0.53	16
South group	80	80	0.61	17

Note

*H*_e_, Nei's genotypic diversity corrected for sample size; MLG, number of diploid individuals multilocus genotypes after clone correction; *N*, total number of samples per collection site; Pa, number of private alleles in each collection site.

### 3.2 Population structure

**Figure 3:**
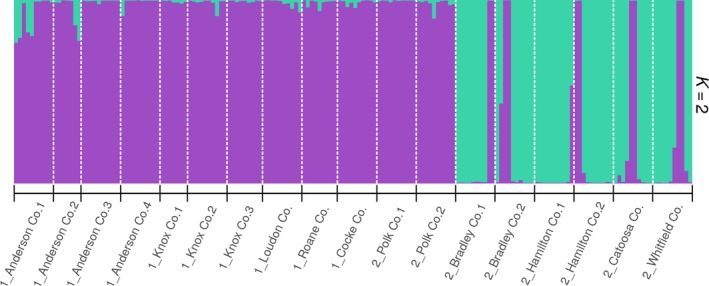
STRUCTURE bar graph representing two genetic clusters (Δ*K* = 2) among 18 collection sites of *Cercis canadensis*. Each vertical bar represents an individual sample, and the color of the bar indicates the assignment probability of that individual to belong to one of the two identified clusters (designated by different colors). The geographical groups are designated as 1 for the north group and 2 for the south group in collection sites label in *X*‐axis

Using the Bayesian clustering analysis, STRUCTURE results indicated an optimum of *∆K* = 2, suggesting that there were two major clusters among 18 collection sites of *C. canadensis* (Figure 3). All collection sites from the north group, along with the collection sites Polk Co.1 (TN) and Polk Co. 2 (TN) of the south group, belonged within the first STRUCTURE‐inferred cluster. The remaining six collection sites from the south group placed in the second inferred cluster. Also, the collection sites of the north group displayed approximately 4% of admixture (less than 80% proportion of any cluster origin). Analysis with an alternative Bayesian algorithm, InStruct (deviance information criterion‐based) further supported our STRUCTURE results. Both Bayesian clustering methods congruently estimated *∆K* = 2 (Figure S2), indicating that there were two genetically distinct clusters present among the studied 18 *C. canadensis* collection sites.

**Figure 4:**
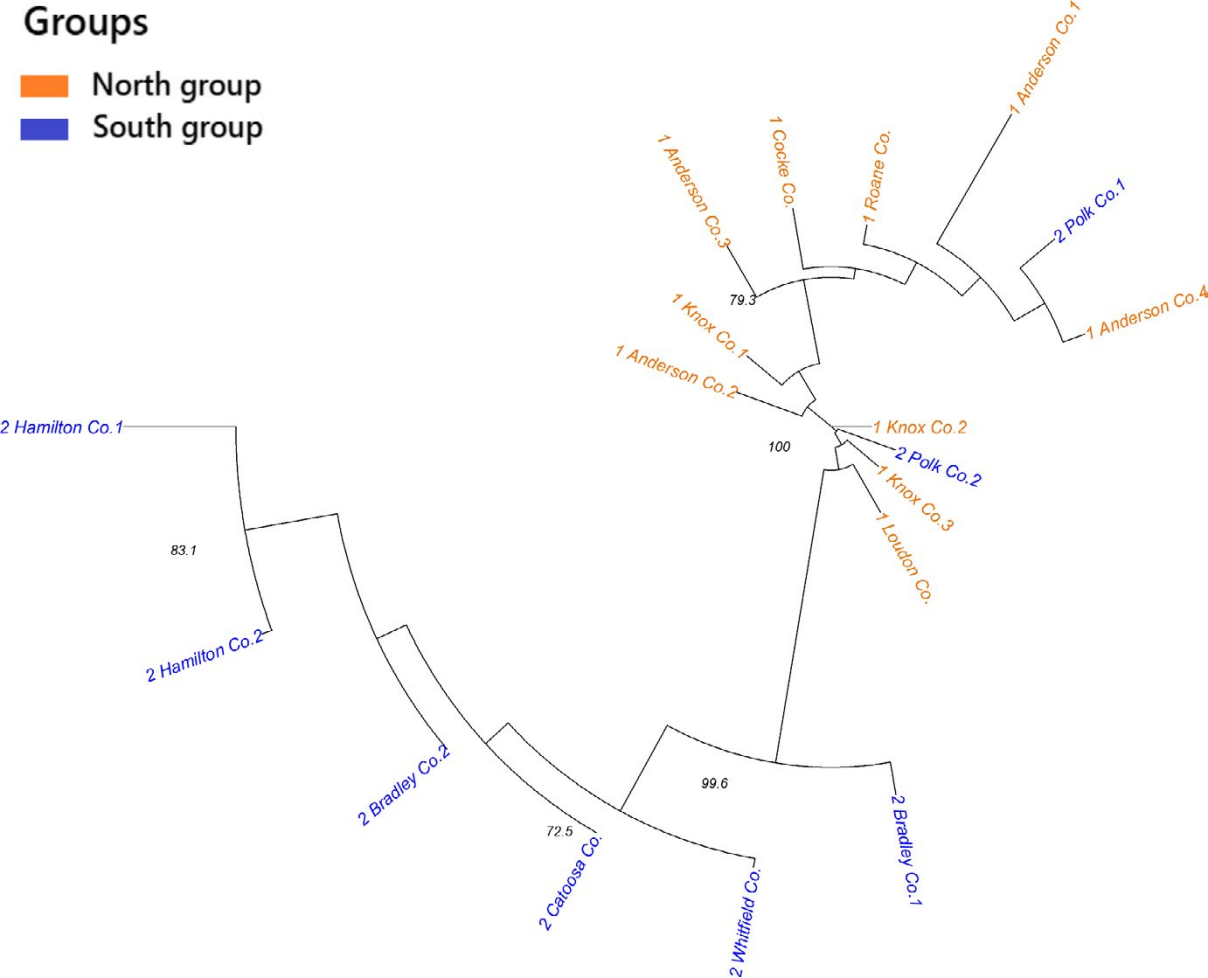
Neighbor‐joining tree of 18 collection sites of *Cercis canadensis* (constructed and visualized using Nei's genetic distance). The geographical groups are designated as “1” for the north group and “2” for the south group in labels. Numbers indicate the percentage of bootstrap support using 1,000 replications

Construction of a neighbor‐joining dendrogram yielded presence of two major clades that were congruent with the STRUCTURE results. In the dendrogram plot, Polk Co.1 and Polk Co.2 from the southern geographical group were placed with the collection sites from the north group (TN; Figure 4). The remaining six collection sites of the south group closely grouped into one clade. Therefore, with exception of two collection sites (Polk Co. 1 and Polk Co. 2 from the south group), collection sites with close geographical proximity belong to the same genetic clusters (Figure 4).

**Figure 5:**
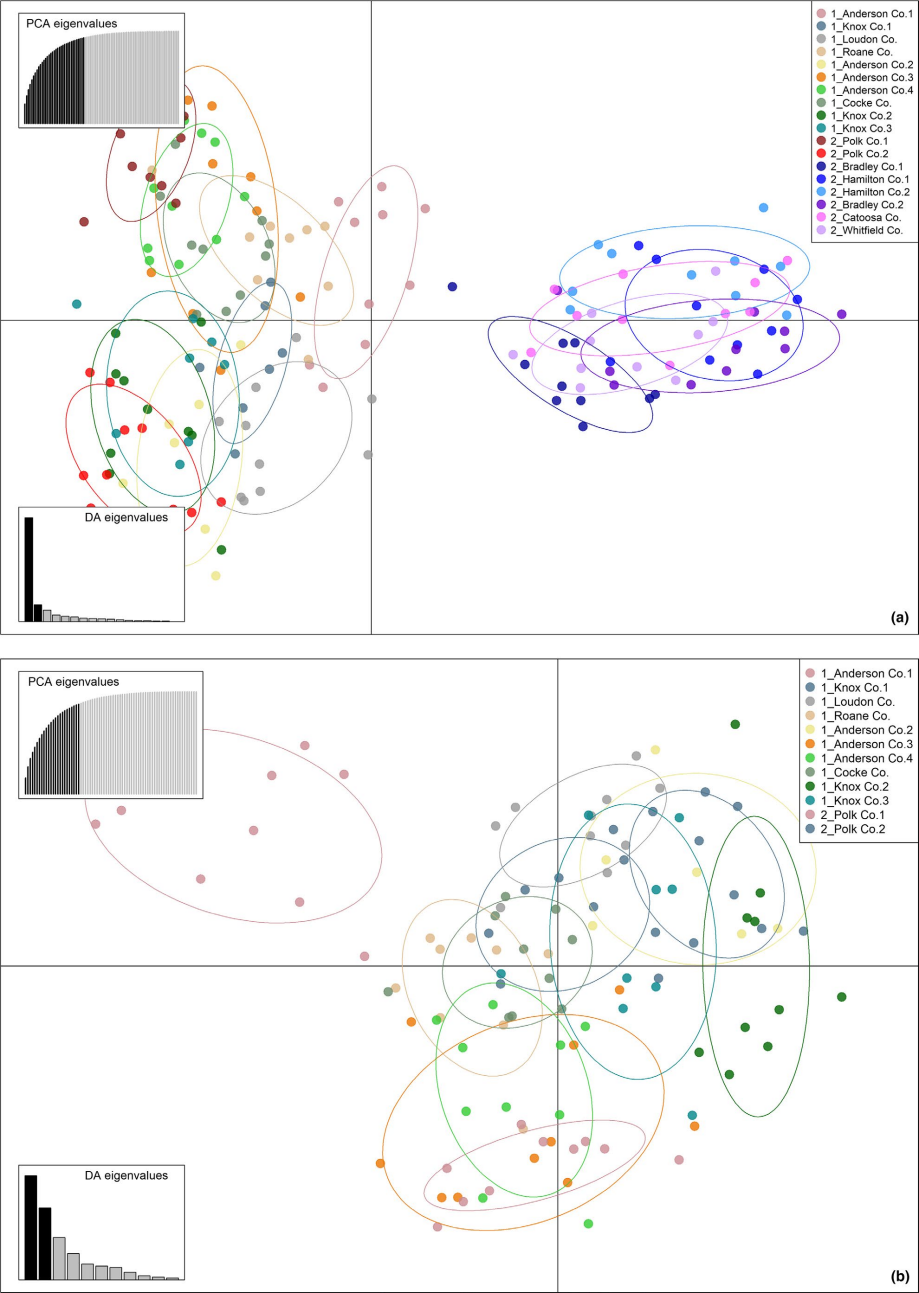
Discriminant analysis of principal components (DAPC) plots of *Cercis canadensis* individuals with 18 collection sites (a) and 12 collection sites (b). In plot A, the first 35 principal components explained 97.3% of the variation in* C. canadensis* individuals from all 18 collection sites. In plot A, allele 149 at locus 680a explained 9.55% of the variance and allele 95 at locus 220a explained 7.03% of the variance on the first axis (threshold = 0.07). In plot B, the first 24 principal components explained 87.8% of the variation in *C. canadensis* individuals from the 12 collection sites. In plot B, allele 108 at locus 220a explained 24.36% of the variance and allele 95 at locus 220a explained 11.51% of the variance on the first axis (threshold = 0.07). Datasets were cross‐checked using 1,000 permutations. Discriminant Analysis (DA) eigenvalues are also presented in the plots

The DAPC plot for 15 microsatellite loci revealed a similar clustering pattern as observed in the STRUCTURE results. The 18 collection sites were divided into two major clusters (Figure 5a). All the collection sites from the south group except Polk Co. 1 (TN, south group) and Polk Co. 2 (TN, south group) were genetically close (similar to the other cluster analysis) and part of a compact cluster. All 10 collection sites from the north group, along with the two collection sites from south group (Polk Co. 1 and Polk Co. 2), were part of another cluster that displayed a wider range of genetic variation than the first cluster (Figure 5a). When 12 collection sites in the second cluster were analyzed independently, the DAPC plot indicated existence of a substructure among those collection sites. Also, individuals from Anderson Co. 1 (TN, north group) were separated by the first axis from all other collection sites (Figure 5b).

The analysis of molecular variance (AMOVA) was tested in the following groupings: (a) 18 collection sites into two major clusters on the basis of the STRUCTURE results and (b) 18 collection sites into two geographical groups (north and south; Table 3). When partitioned by two clusters according to STRUCTURE results, only 27.85% (*p* < .001) of the variation was present between the clusters, whereas 7% (*p* < .001) of the variation was attributed among collection sites within these two clusters (Table 3). The greatest level of variance was partitioned among two clusters—65.14% (*p* < .001). When the data were divided into two geographical groups (north and south), 14.88% (*p* < .001) of observed variation occurred between the groups, whereas only 14.33% (*p* < .001) of the variation was attributed among collection sites within these two groups (Table 3). The vast majority of the variance was present among individuals within collection sites (70.79%, *p* < .001; Table 3).

**Table 3:** Analysis of molecular variance (AMOVA) of *Cercis canadensis* across 15 microsatellite loci for (a) 18 collection sites into two groups according to two clusters of STRUCTURE and (b) 18 collection sites as two groups (north and south groups)

Source of variations	*df*	Sum of squares	Variance	% Variation
(a) Two clusters (STRUCTURE)				
Among clusters	1	154.07	1.86	27.85
Among collection sites within clusters	16	140.78	0.47	7.01
Within collection sites	154	669.10	4.34	65.14
Total	171	963.96	6.67	100.00
*F*_ST_ = 0.35, *F*_SC_ = 0.1, *F*_CT_ = 0.28				
(b) Two groups (north and south groups)				
Among south and north groups	1	91.04	0.92	14.88
Among collection sites within two groups	16	203.82	0.88	14.33
Within collection sites	154	669.10	4.34	70.79
Total	171	963.96	6.14	100
*F*_ST_ = 0.29, *F*_SC_ = 0.17, *F*_CT_ = 0.15				

Note

*F*_ST_ = variance among collection sites relative to the total variance. *F*_SC_ = variance among collection sites within groups. *F*_CT_ = variance among groups relative to the total variance.

Isolation‐by‐distance analysis suggested that the geographical distance was linearly correlated (*r* = .39, *p* < .001) with genetic distance of the sampled data (Figure S3). However, when analyzed separately by geographical groups, there was no evidence of correlation between genetic and geographical distance among *C. canadensis* individuals (north group *r* = −.03, *p* = .70; south group *r* = −.07, *p* = .14).

### 3.3 Population demography

Using the BOTTLENECK software with S.M.M., I.A.M., and T.P.M., data were partitioned and analyzed based on STRUCTURE results. Sign tests revealed no significant excess of heterozygosity among *C. canadensis* collection sites for the analyzed loci. Also, the results indicated a normal L‐shaped allele frequency distribution observed in both clusters, indicating that *C. canadensis* collection sites from eastern Tennessee and along the Georgia–Tennessee border have not been subjected to any recent bottleneck events (Table S3).

## 4 DISCUSSION

Long‐term evolutionary success and ultimately species survival is influenced by population size, genetic diversity, allelic richness, fitness, and substantial gene flow, which are of fundamental importance in plant ecology, evolution, and conservation (Leimu, Mutikainen, Koricheva, & Fischer, 2006). Our study, which is part of a larger population assessment effort for *C. canadensis* in the United States, has revealed high levels of genetic diversity and allelic richness, moderate genetic differentiation, presence of genetic structure, and high gene flow between *C. canadensis* wild populations distributed within fragmented forest patches in eastern Tennessee and along the Georgia–Tennessee border. These results are congruent with other studies of temperate tree species, suggesting that populations of woody forest trees maintain high levels of genetic diversity at the population level compared to herbaceous plants (Chang, Bongarten, & Hamrick, 1998; Hamrick & Godt, 1996; Marquardt & Epperson, 2004; Victory et al., 2006).

Many forest areas in eastern North America are affected negatively by human disturbances (Foster, Motzkin, & Slater, 1998; LaBonte, Tonos, Hartel, & Woeste, 2017), insect and pathogen infestations (Orwig & Foster, 1998; Ramsfield, Bentz, Faccoli, Jactel, & Brockerhoff, 2016; Trumbore, Brando, & Hartmann, 2015), drought (Allen, Breshears, & McDowell, 2015; Klos, Wang, Bauerle, & Rieck, 2009; Millar & Stephenson, 2015), wildfire (Mutch et al., 1993), and global climate change (Trumbore et al., 2015). Human disturbances in forest ecosystems include land conversion for agriculture and settlements (Foster et al., 1998; Weir & Ott, 1997), logging (Hayes, Moody, White, & Costanza, 2007; Pyle, 1984), and fragmentation by building infrastructure (Kwak et al., 1998). In the 1800s and 1900s, human disturbances greatly affected the forest area in the southern Appalachian region and altered the ecosystem by logging, forest clearing, and burning, thus creating poor soil conditions and erosion (McLaughlin, Andersen, Hanson, Tjoelker, & Roy, 1991; Pyle, 1984; Wear & Greis, 2002; White, Gevel, & Soulé, 2012). Despite anthropogenic disturbances and habitat fragmentation, many forest trees were able to maintain high genetic diversity at population levels (Brunet, Zalapa, & Guries, 2016; Chang et al., 1998; Hamrick & Godt, 1996; Hamrick, Godt, & Sherman‐Broyles, 1992; Marquardt & Epperson, 2004; Nybom, 2004; Petit & Hampe, 2006; Wang et al., 2014). This resilience can be explained in part by species biology, including (but not limited to) widespread distribution, outcrossing mating systems, high level of gene flow due to long distance pollen or seed dispersal, and presence of suitable population size (Dubreuil et al., 2010; Hamrick et al., 1992; Petit & Hampe, 2006).

*Cercis canadensis* populations examined in this study had higher genetic differentiation when compared to other hardwood species such as *Cornus florida* L. (*F*_ST_ = 0.07), *Cunninghamia lanceolata* (Lamb.) Hook.] (*F*_ST_ = 0.04), and *Viburnum rufidulum* Raf. (*F*_ST_ = 0.06) (Dean et al., 2015; Duan et al., 2015; Hadziabdic et al., 2012; Hamrick et al., 1992; Hardesty, Dick, Kremer, Hubbell, & Bermingham, 2005). Similar to findings of this report, understory temperate trees species, *Sorbus torminalis*L. (Crantz) (wild service tree, *F*_ST_ = 0.17) and *Cladrastis kentukea* (Dum. Cours.) Rudd (Yellowwood, *F*_ST_ = 0.11–0.23), showed strong genetic differentiation, which was likely related to their patchy distributions and population structure (Bednorz & Kosiński, 2006; LaBonte et al., 2017). Unlike *S. torminalis* and *C. kentukea*, *C. canadensis* is widely distributed across the United States and found in a wide range of ecological habitats. Therefore, high genetic differentiation is probably related to tree reproductive biology, as well as a combination of other factors, including dispersal method and local isolations.

Based on our study results, we reject the hypothesis of limited gene flow as a result of increased distance between the examined groups of *C. canadensis.* Limited gene flow typically leads to reduced genetic diversity, increased population structure, and inbreeding within populations (Byrne et al., 2008; Sherwin & Moritz, 2000; Young et al., 1996), which were not consistent with our findings. Other studies focused on forest trees in fragmented landscapes found that regardless of moderate‐to‐high habitat fragmentation, many insect‐pollinated tree species were able to maintain high levels of gene flow across isolated patches through increased long distance pollen dispersal (Bacles, Burczyk, Lowe, & Ennos, 2005; Colabella, Gallo, Moreno, & Marchelli, 2014; Nason & Hamrick, 1997; Wang, Stephen, & Xiao‐Yong, 2011). Our study findings are consistent with these studies as shown by the high level of gene flow present across fragmented *C. canadensis* populations. Therefore, forest fragmentation did not negatively influence the gene flow across isolated populations of *C. canadensis* in the eastern Tennessee and Georgia–Tennessee border.

Eastern redbud is similar to many other self‐incompatible species (Roberts et al., 2015), wherein *C. canadensis* depends on insect‐, mammal‐, and bird‐mediated pollination for dispersal. In its natural distribution pollinators of *C. canadensis* include honeybees, megachilid bees, small sweat bees, butterflies, and beetles (Dickson, 1990; Kraemer & Favi, 2010; Tucker, 2002). These insects are usually capable of flying one to several kilometers, depending on the insect species and environmental conditions, so pollen‐based gene flow was expected to be high across fine‐scale geographical ranges (Hagler, Mueller, Teuber, Machtley, & Deynze, 2011; Kramer et al., 2008; Pasquet et al., 2008). In a large, continuous forest area, the majority of the pollination that occurs by insects is among neighboring trees. In contrast, with increasing distance between isolated patches, pollen source in a fragmented area can become rather difficult to obtain, further facilitating insufficient pollen dispersal in these fragmented patches (Dick, Etchelecu, & Austerlitz, 2003; Lowe et al., 2003; Sork & Smouse, 2006; White, Boshier, & Powell, 1999).

Although pollen dispersal by insects could be a primary medium of gene flow (Vekemans & Hardy, 2004), a substantial amount of *C. canadensis* seed movement depends on birds and other small mammals. The gene flow introduced via animal‐mediated seed dispersal is therefore dependent upon the dispersal mode and behavior patterns of the animals that forage on the fruits and eat the seeds. Heavy *C. canadensis* fruits (pods) harbor seeds with a hard testa that usually fall in close proximity to the parent tree and then germinate within a year or two (Dickson, 1990; Hayden, 2013). Hence, some of the seedlings could grow in close proximity to the mother tree, which could potentially create half‐sib neighborhoods on a small spatial scale (Gonzales, Hamrick, Smouse, Trapnell, & Peakall, 2009; Nakanishi, Tomaru, Yoshimaru, Manabe, & Yamamoto, 2008; Schnabel, Laushman, & Hamrick, 1991; Vekemans & Hardy, 2004). Assessments of pollen or seed dispersal patterns in *C. canadensis* were beyond the scope of this study; therefore, we can only provide this as a plausible explanation for our findings. An alternative explanation for presence of the half‐sib neighbors is that fruits are also ingested by small rodents (e.g., gray squirrel, eastern woodrat), white‐tailed deer, quail, pheasants, and several other bird species which are then dispersed in scat (Dickson, 1990; Post, 1992; Wakeland & Swihart, 2009; Wright, Fleming, & Post, 1990). Related individuals (from seeds that originated with the same mother tree) can be carried to a nearby location by an individual animal feeding on the fruits from the same mother tree. For example, the hoarding behavior of the rodents helps related propagules to be dispersed to a close‐proximity destination (Post, 1992; Setoguchi, 1990). On the other hand, birds occasionally ingest *C. canadensis* seeds and can dispose of them in new localities across longer distances, resulting in an increased gene flow among the populations (Hadziabdic et al., 2012; Sullivan, 1994). The direction and rate of dispersal of such *C. canadensis* seeds would depend on the foraging behavior of the seed carrier and the ecological conditions of their habitat.

Although animals may occasionally choose *C. canadensis* seed pods as a food source during summer and winter months when other food resources are scarce (Post, 1992; Short & Epps, 1976), this is less common scenario for seed dispersal (Dickson, 1990; Halls & Crawford, 1960; Wakeland & Swihart, 2009). Moreover, *C. canadensis* seed dispersal that is mostly animal‐dependent can become more limiting within isolated areas, further increasing half‐sibling mating and structuring among *C. canadensis* populations (Koprowski, 2005). Therefore, our hypothesis was that spatial genetic structure of *C. canadensis* could be influenced by the forest fragmentation despite the presence of pollen‐mediated gene flow in the small geographical location (Chung, Nason, Epperson, & Chung, 2003; Wang et al., 2011). Furthermore, our study revealed significant isolation by distance in local populations of *C. canadensis* and indicated that genetic distance between *C. canadensis* populations was significantly correlated with the geographical distance between them. Therefore, geographical distance or barriers may influence spatial genetic structure in *C. canadensis* populations. Additional research undertaken to evaluate pollen and seed dispersal methods and distances covered by seed carriers would help articulate the gene flow mechanisms for this native tree species.

Our results indicated high genetic diversity and high gene flow, which suggests reproductive isolation of *C. canadensis* caused by fragmentation may not be of great concern for current populations. These findings are congruent with other population genetics studies of temperate, self‐incompatible tree species. Also, current isolated patches and remnant populations are genetically stable allowing for maintenance of viable populations at a geographically fine‐scale level. Although natural stands of *C. canadensis* seemingly maintain genetically fit populations, we cannot rule out the possible negative effect of forest fragmentation on *C. canadensis* population viability. As this is an outcrossing, self‐incompatible, and animal‐dispersed tree species, limitation or reduction in the number of seed dispersal agents (e.g., small rodents) in these fragmented populations can affect the fitness of *C. canadensis* populations in the future. Therefore, it is important to ensure that healthy and diverse habitats are present for animals responsible for seed and pollen dispersal of *C. canadensis*. Also, to better understand the effect of habitat fragmentation on *C. canadensis*, we suggest further studies be conducted on its sexual reproduction and life‐history traits.

Over the past few decades, a number of studies have been conducted to better understand the genetic consequence of habitat fragmentation and anthropogenic disturbances on rare forest species. However, very few studies have been conducted to evaluate the effects of these disturbances on common forest species (Aguilar, Ashworth, Galetto, & Aizen, 2006). Our study showed the consequences of recent habitat fragmentations on an economically important and widely distributed native species *C. canadensis*. We suggest that despite the current resilient genetic diversity and high evolutionary potential, *C. canadensis* populations and other species in the fragmented habitats may suffer severe consequences following further environmental changes and climate threats. Therefore, to better understand the potential consequences of these threats, research on the population genetics of other concurring species is necessary for conservation efforts and habitat management of forest ecosystems. Our results also suggested that wild populations of *C. canadensis* with high genetic variations can be used as reservoirs of desired genetic variations and can be utilized in breeding programs to improve phenotypic traits in the nursery stocks.

## CONFLICT OF INTEREST

None declared.

## AUTHORS' CONTRIBUTION

DH, WK, and RT conceived and planned the experiments including the main conceptual ideas and proof outline. MO and SB carried out the experiments, MO, MN, SB, and DH troubleshot technical details. All authors contributed to sample collection and preparation. MO, MN, JZ, and DH contributed to data analyses. All authors contributed to the interpretation of the results, manuscript writing, and editing. All authors provided critical feedback and helped shape the research, analysis, and manuscript.

## Supporting information

Supinfo

## Data Availability

Ony et al. (2020) The effect of habitat fragmentation on genetic diversity and differentiation: Fine‐scale population structure of *Cercis canadensis* (eastern redbud), Dryad, Dataset: https://doi.org/10.5061/dryad.59zw3r243.
