# Supplementary material for: Habitat fragmentation influences genetic diversity and differentiation: Fine‐scale population structure of Cercis canadensis (eastern redbud)
Source: Ecol Evol. 2020 Mar 16;10(8):3655–70. doi: 10.1002/ece3.6141 (PMC7160182; doi:10.1002/ece3.6141)
Supplement: Supplementary file 1 — Supinfo [file ECE3-10-3655-s001.docx]

Table S1. Eighteen collection sites of *Cercis canadensis* collected from Tennessee and Georgia in the eastern United States.

| **Sampling State** | **Collection Site** | **Number of Individuals** | **Group** | **Longitude** | **Latitude** |
| --- | --- | --- | --- | --- | --- |
| Tennessee | Anderson Co.1 | 10 | North Group | 36.04767 | -84.20664 |
| Tennessee | Knox Co.1 | 10 | North Group | 35.95661 | -84.16416 |
| Tennessee | Loudon Co. | 10 | North Group | 35.88551 | -84.32235 |
| Tennessee | Roane Co. | 10 | North Group | 35.97289 | -84.34798 |
| Tennessee | Anderson Co.2 | 10 | North Group | 36.03032 | -84.18986 |
| Tennessee | Anderson Co. 3 | 10 | North Group | 36.02337 | -84.18487 |
| Tennessee | Anderson Co. 4 | 10 | North Group | 35.99935 | -84.21875 |
| Tennessee | Cocke Co. | 10 | North Group | 35.82051 | -83.14966 |
| Tennessee | Knox Co.2 | 10 | North Group | 36.04097 | -83.93403 |
| Tennessee | Knox Co.3 | 10 | North Group | 35.94648 | -83.91469 |
| Tennessee | Polk Co. 1 | 10 | South Group | 35.15252 | -84.60653 |
| Tennessee | Polk Co. 2 | 10 | South Group | 35.18276 | -84.43995 |
| Tennessee | Bradley Co.1 | 10 | South Group | 35.12912 | -85.01062 |
| Tennessee | Hamilton Co.1 | 10 | South Group | 35.08390 | -85.11900 |
| Tennessee | Hamilton Co.2 | 10 | South Group | 35.02323 | -85.37941 |
| Tennessee | Bradley Co.2 | 10 | South Group | 35.12999 | -84.73290 |
| Georgia | Catoosa Co. | 10 | South Group | 34.90867 | -85.10970 |
| Georgia | Whitfield Co. | 10 | South Group | 34.73177 | -85.03349 |

Table S2. Pairwise *F_ST_* matrix of Nei's genetic distance of *Cercis canadensis* utilizing 18 collection sites.

| Collection Sites | 1_Anderson Co.1 | 1_Knox Co.1 | 1_Loudon Co. | 1_Roane Co. | 1_Anderson Co.2 | 1_Anderson Co.3 | 1_Anderson Co.4 | 1_Cocke Co. | 1_Knox Co.2 | 1_Knox Co.3 | 2_Polk Co.1 | 2_Polk Co.2 | 2_Bradley Co.1 | 2_Hamilton Co.1 | 2_Hamilton Co.2 | 2_Bradley Co.2 | 2_Catoosa Co. |
| --- | --- | --- | --- | --- | --- | --- | --- | --- | --- | --- | --- | --- | --- | --- | --- | --- | --- |
| 1_Knox Co.1 | 0.12 |  |  |  |  |  |  |  |  |  |  |  |  |  |  |  |  |
| 1_Loudon Co. | 0.13 | 0.07 |  |  |  |  |  |  |  |  |  |  |  |  |  |  |  |
| 1_Roane Co. | 0.08 | 0.06 | 0.11 |  |  |  |  |  |  |  |  |  |  |  |  |  |  |
| 1_Anderson Co.2 | 0.12 | 0.06 | 0.08 | 0.07 |  |  |  |  |  |  |  |  |  |  |  |  |  |
| 1_Anderson Co.3 | 0.10 | 0.08 | 0.10 | 0.06 | 0.10 |  |  |  |  |  |  |  |  |  |  |  |  |
| 1_Anderson Co.4 | 0.08 | 0.10 | 0.14 | 0.05 | 0.11 | 0.06 |  |  |  |  |  |  |  |  |  |  |  |
| 1_Cocke Co. | 0.09 | 0.06 | 0.11 | 0.05 | 0.09 | 0.05 | 0.05 |  |  |  |  |  |  |  |  |  |  |
| 1_Knox Co.2 | 0.15 | 0.06 | 0.06 | 0.10 | 0.05 | 0.07 | 0.12 | 0.08 |  |  |  |  |  |  |  |  |  |
| 1_Knox Co.3 | 0.13 | 0.06 | 0.04 | 0.08 | 0.06 | 0.09 | 0.11 | 0.08 | 0.04 |  |  |  |  |  |  |  |  |
| 2_Polk Co.1 | 0.11 | 0.12 | 0.17 | 0.05 | 0.13 | 0.07 | 0.04 | 0.07 | 0.15 | 0.13 |  |  |  |  |  |  |  |
| 2_Polk Co.2 | 0.15 | 0.08 | 0.07 | 0.12 | 0.05 | 0.11 | 0.13 | 0.11 | 0.04 | 0.05 | 0.17 |  |  |  |  |  |  |
| 2_Bradley Co.1 | 0.17 | 0.13 | 0.12 | 0.17 | 0.13 | 0.16 | 0.21 | 0.15 | 0.12 | 0.11 | 0.24 | 0.13 |  |  |  |  |  |
| 2_Hamilton Co.1 | 0.23 | 0.20 | 0.20 | 0.24 | 0.19 | 0.24 | 0.28 | 0.23 | 0.19 | 0.19 | 0.31 | 0.19 | 0.06 |  |  |  |  |
| 2_Hamilton Co.2 | 0.18 | 0.16 | 0.16 | 0.19 | 0.17 | 0.18 | 0.23 | 0.17 | 0.15 | 0.16 | 0.26 | 0.17 | 0.06 | 0.04 |  |  |  |
| 2_Bradley Co.2 | 0.15 | 0.14 | 0.15 | 0.17 | 0.14 | 0.17 | 0.20 | 0.16 | 0.14 | 0.14 | 0.23 | 0.14 | 0.06 | 0.04 | 0.05 |  |  |
| 2_Catoosa Co. | 0.15 | 0.13 | 0.14 | 0.15 | 0.14 | 0.16 | 0.20 | 0.15 | 0.13 | 0.12 | 0.22 | 0.14 | 0.05 | 0.05 | 0.04 | 0.02 |  |
| 2_Whitfield Co. | 0.15 | 0.13 | 0.14 | 0.17 | 0.14 | 0.17 | 0.21 | 0.15 | 0.12 | 0.13 | 0.24 | 0.13 | 0.05 | 0.04 | 0.04 | 0.03 | 0.02 |

Table S3. Bottleneck determination by sign tests for *Cercis canadensis* samples utilizing 15 microsatellite loci.

| **Mutation model (excess/deficit) ^a^** | | | | | |
| --- | --- | --- | --- | --- | --- |
| **Cluster** | **I.A.M** | **T.P.M** | **S.M.M** | **Mode-shift^b^** | ***P* value** |
| **Cluster 1** | 10/5 | 4/11 | 0/15 | Not shifted | NS |
| **Cluster 2** | 13/3 | 9/6 | 4/11 | Not shifted | NS |

I.A.M. = infinite allele model; T.P.M. = two-phase mutation model; S.M.M. = stepwise mutation model; a = Excess/deficit indicates the number of loci showing excess/deficit of gene diversity under mutation-drift in populations that experienced a recent bottleneck; b = A shift in the distribution of allelic frequency classes is expected in populations that experienced a recent bottleneck; NS = not significant


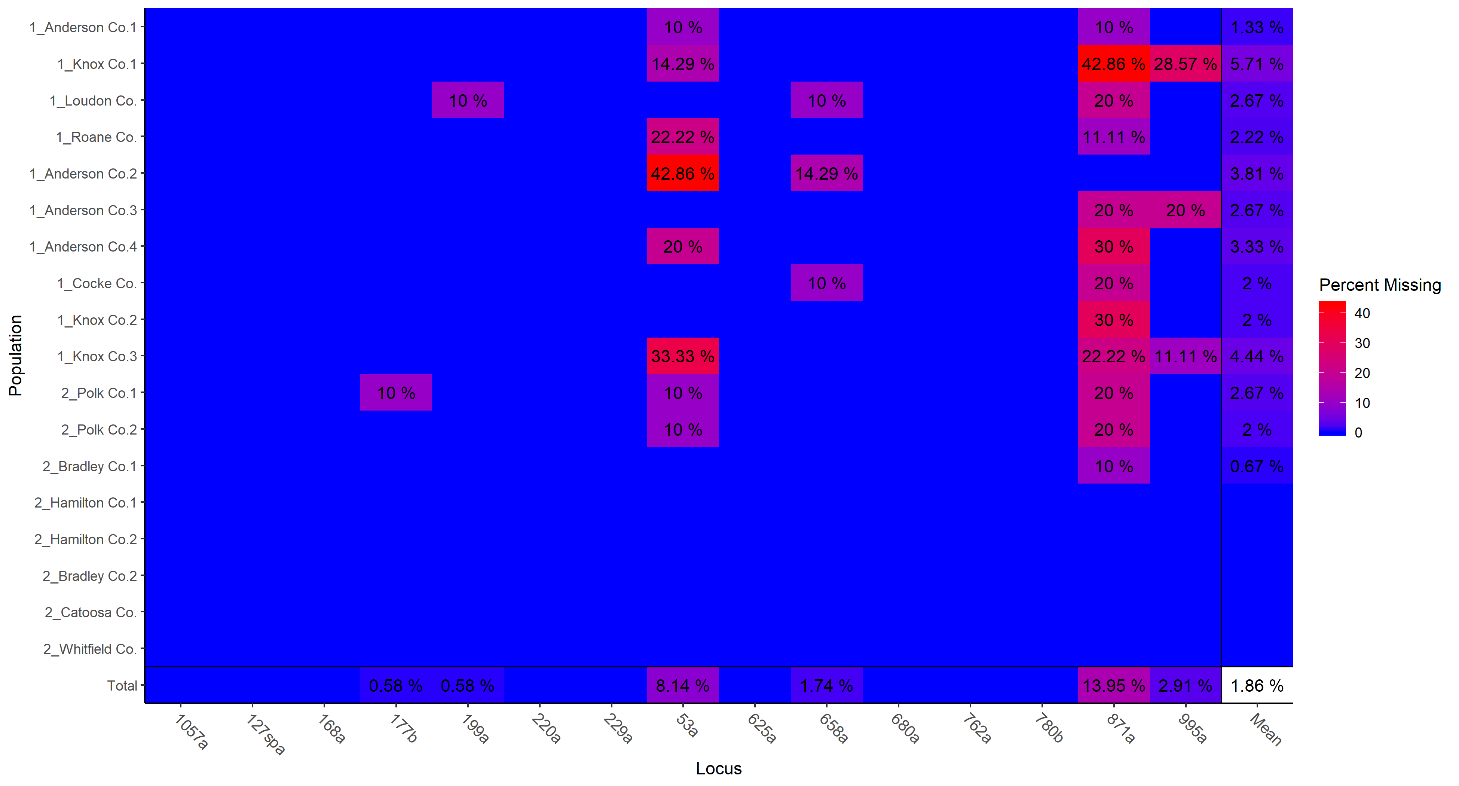


Figure S1. Plot representing missing values of *Cercis canadensis* dataset across 15 microsatellite loci (locus) and 18 collection sites (population).


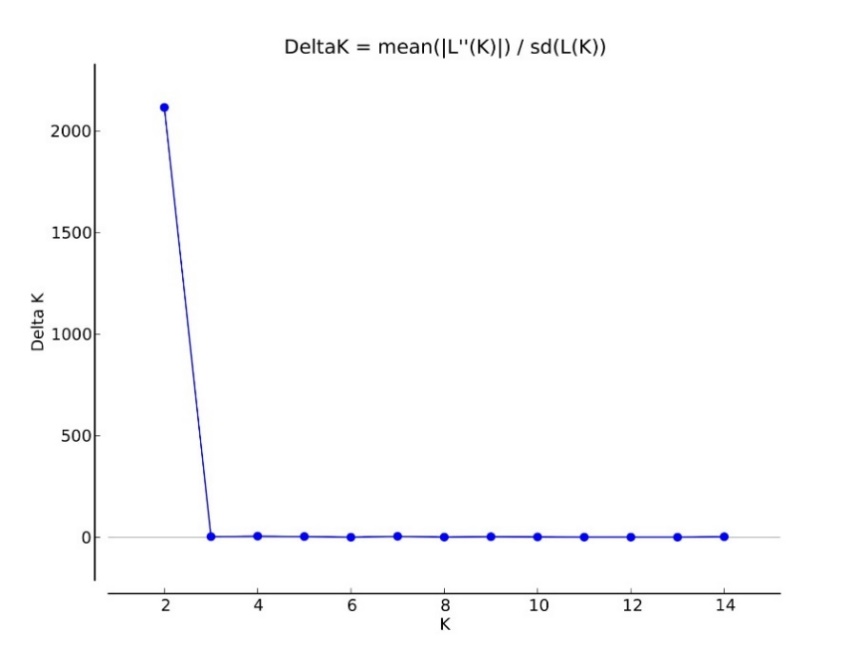


Figure S2. Bayesian clustering probabilities using programs STRUCTURE and InStruct. STRUCTURE results were obtained using *∆K*, with graph results indicating two genetic clusters among 18 *Cercis canadensis* collection sites (A). InStruct results were obtained using deviance information criterion calculations (B). Results suggested two different genetic clusters (*K* = 2) across all 18 *Cercis canadensis* collection sites.


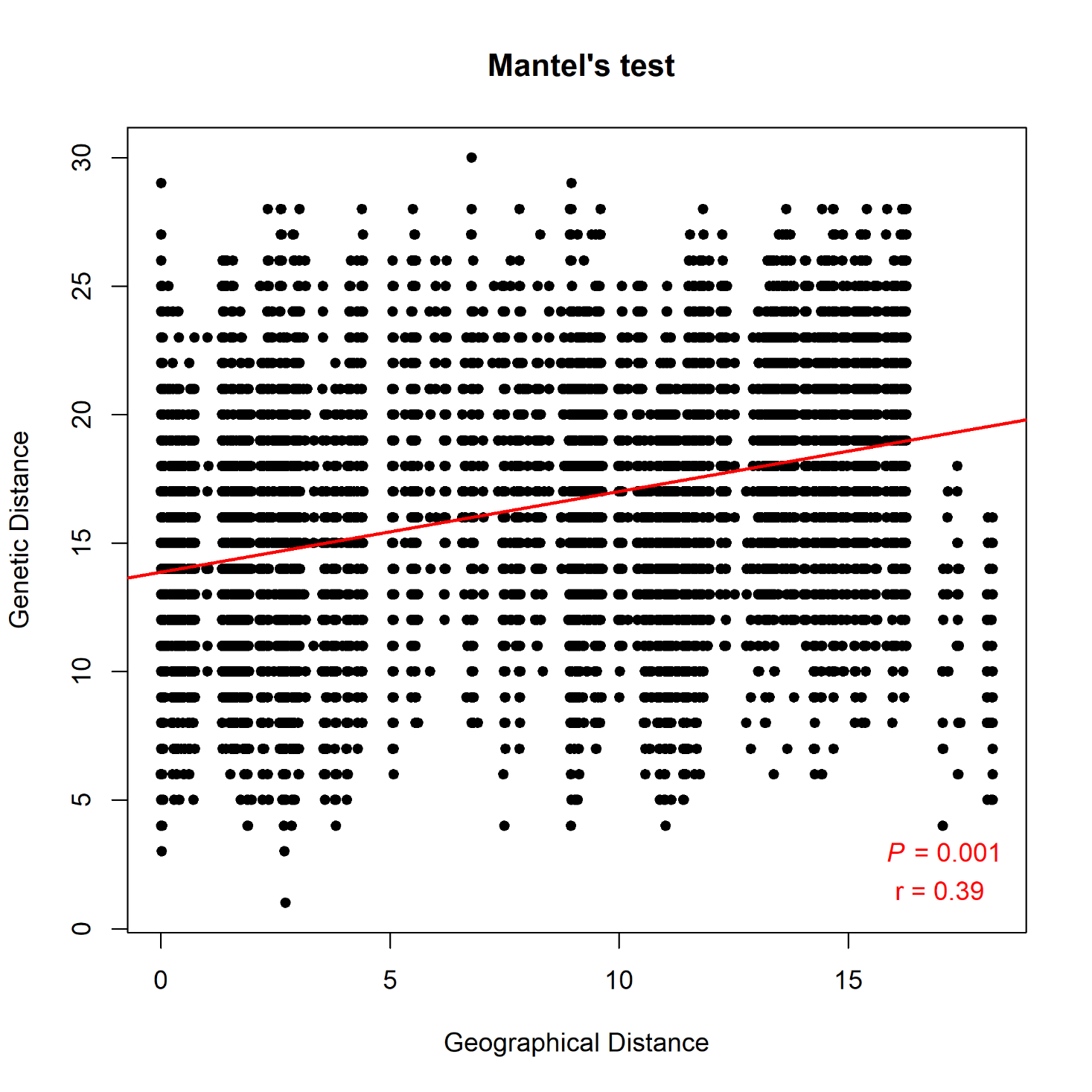


Figure S3. Mantel test of *Cercis canadensis* individuals across 18 collection sites in eastern Tennessee and around the Georgia-Tennessee border. This figure represents the correlation analysis between geographical distance and genetic distance.
